# Two New Fatty Acid Derivatives, Omphalotols A and B and Anti-*Helicobacter pylori* Fatty Acid Derivatives from Poisonous Mushroom *Omphalotus japonicus*

**DOI:** 10.3390/ph15020139

**Published:** 2022-01-25

**Authors:** Seulah Lee, Tae Wan Kim, Yong Hoon Lee, Dong-Min Kang, Rhim Ryoo, Yoon-Joo Ko, Mi-Jeong Ahn, Ki Hyun Kim

**Affiliations:** 1 School of Pharmacy, Sungkyunkwan University, Suwon 16419, Korea; seulah@kopri.re.kr (S.L.); asde8282@naver.com (T.W.K.); yhl2090@naver.com (Y.H.L.); 2 Division of Life Sciences, Korea Polar Research Institute, KIOST, Incheon 21990, Korea; 3 College of Pharmacy and Research Institute of Pharmaceutical Sciences, Gyeongsang National University, Jinju 52828, Korea; kdm7105@gnu.ac.kr (D.-M.K.); amj5812@gnu.ac.kr (M.-J.A.); 4 Special Forest Products Division, Forest Bioresources Department, National Institute of Forest Science, Suwon 16631, Korea; rryoo@korea.kr; 5 Laboratory of Nuclear Magnetic Resonance, National Center for Inter-University Research Facilities (NCIRF), Seoul National University, Gwanak-gu, Seoul 08826, Korea; yjko@snu.ac.kr

**Keywords:** *Omphalotus japonicus*, Marasmiaceae, fatty acid derivatives, LC–MS/MS, anti-*H. pylori* activity

## Abstract

As part of ongoing systematic research into the discovery of bioactive secondary metabolites with novel structures from Korean wild mushrooms, we investigated secondary metabolites from a poisonous mushroom, *Omphalotus japonicus* (Kawam.) Kirchm. & O. K. Mill. belonging to the family Marasmiaceae, which causes nausea and vomiting after consumption. The methanolic extract of *O. japonicus* fruiting bodies was subjected to the fractionation by solvent partition, and the CH_2_Cl_2_ fraction was analyzed for the isolation of bioactive compounds, aided by an untargeted liquid chromatography mass spectrometry (LC–MS)-based analysis. Through chemical analysis, five fatty acid derivatives (**1**–**5**), including two new fatty acid derivatives, omphalotols A and B (**1** and **2**), were isolated from the CH_2_Cl_2_ fraction, and the chemical structures of the new compounds were determined using 1D and 2D nuclear magnetic resonance (NMR) spectroscopy and high resolution electrospray ionization mass spectrometry (HR-ESIMS), as well as fragmentation patterns in MS/MS data and chemical reactions followed by the application of Snatzke’s method and competing enantioselective acylation (CEA). In the anti-*Helicobacter pylori* activity test, compound **1** showed moderate antibacterial activity against *H. pylori* strain 51 with 27.4% inhibition, comparable to that of quercetin as a positive control. Specifically, compound **3** exhibited the most significant antibacterial activity against *H. pylori* strain 51, with MIC_50_ and MIC_90_ values of 9 and 20 μM, respectively, which is stronger inhibitory activity than that of another positive control, metronidazole (MIC_50_ = 17 μM and MIC_90_ = 46 μM). These findings suggested the experimental evidence that the compound **3**, an α,β-unsaturated ketone derivative, could be used as a moiety in the development of novel antibiotics against *H. pylori*.

## 1. Introduction

Mushrooms have been used to treat various diseases in traditional medicine [1], and a number of pharmacological and phytochemical studies on mushrooms have demonstrated that they are rich sources of various bioactive compounds that exhibit beneficial immunomodulatory, antioxidant, and angiostatic activities, as well as cytotoxicity against cancers [1,2,3,4,5]. Based on this evidence, mushrooms have emerged as potential valuable sources of bioactive natural products; however, most studies have focused on medicinal and edible mushrooms, and little is known regarding bioactive secondary metabolites produced from poisonous mushrooms.

As part of ongoing systematic research on Korean wild mushrooms for the discovery of bioactive secondary metabolites with novel structures [6], we investigated bioactive secondary metabolites from a poisonous mushroom; *Omphalotus japonicus* (Kawam.) Kirchm. & O. K. Mill. *O. japonicus* is an orange-to-brown-colored gilled mushroom belonging to the family Marasmiaceae, which is found in Japan and Eastern Asia. It is a member of the genus *Omphalotus*, the members of which have bioluminescent fruit bodies that glow in dark [7]. This poisonous mushroom causes nausea and vomiting after consumption. Sesquiterpenoids have been identified as the major secondary metabolites in *O. japonicus*, the most well-known of which is illudin S, a representative toxic metabolite that exhibits potent cytotoxic and antiviral activities [8]. It has also displayed strong in vitro and in vivo antitumor activity against multi-drug-resistant tumors, and a novel anticancer drug, irofulven, was developed based on the structure and anticancer activity of illudin S [9,10,11,12,13]. Additionally, the potent cytotoxicity of illudin S has extended its application to other pharmacological effects, where its antiviral activity in an HSV-I/CV-1 assay and glutathione reductase inhibition have been confirmed [8,13,14]. Toxic illudane-type sesquiterpenes from *O. japonicus*, including dihydroilludin S and neoilludins A and B, have also been reported [15,16]. As other secondary metabolites, the luminescent substances, lampteroflavin [17], lampterol [18] have been identified from this mushroom, and polysaccharides [19] from *O. japonicus* have been reported to show antitumor activities.

In the present study, we conducted the fractionation of the methanolic extract of *O. japonicus* fruiting bodies, and chemical analysis of CH_2_Cl_2_ fraction was carried out to isolate potential bioactive compounds aided by an untargeted liquid chromatography-tandem mass spectrometry (LC–MS/MS)-based analysis. Five fatty acid derivatives (**1**–**5**), including two new fatty acid derivatives, omphalotols A and B (**1** and **2**), were isolated from the CH_2_Cl_2_ fraction. Herein, we describe the isolation and structural determination of compounds **1**–**5,** and evaluate their anti-*H. pylori* activity.

## 2. Results and Discussion

### 2.1. Extraction of O. japonicus and Isolation of Compounds

**Figure 1:**
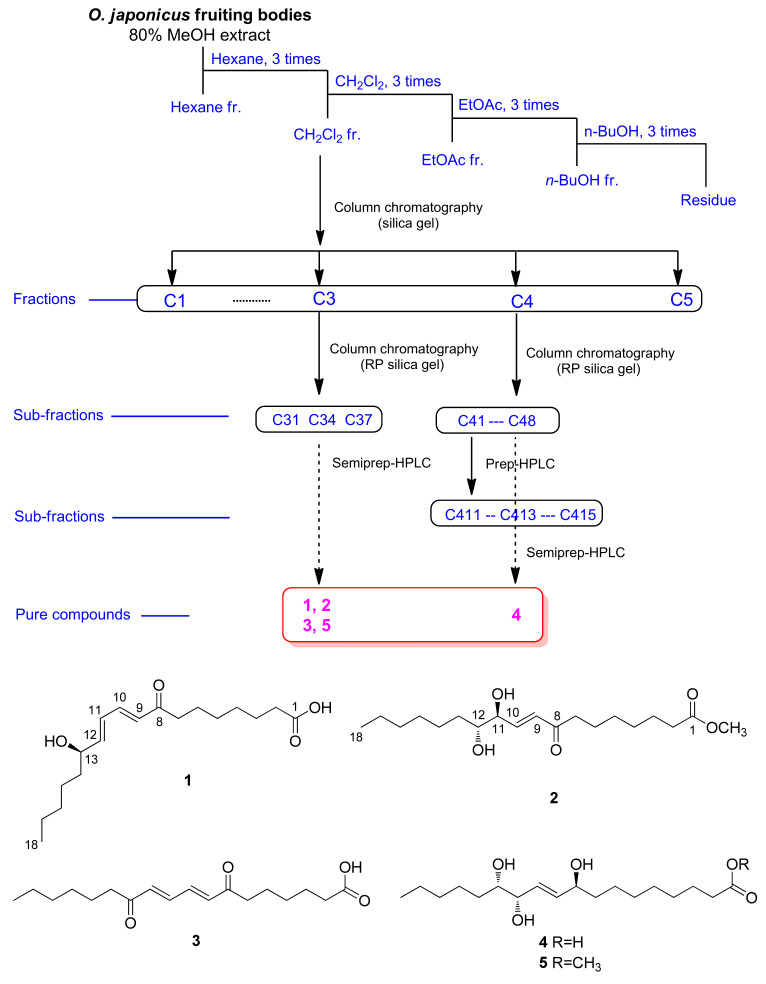
Separation scheme (**top**) and chemical structures (**bottom**) of compounds **1**–**5**.

Dried *O. japonicus* fruiting bodies were extracted with 80% methanol, and the crude methanolic extract was extracted by rotary evaporation. The resultant MeOH extract was sequentially applied to solvent partitioning using *n*-hexane, dichloromethane (CH_2_Cl_2_), ethyl acetate (EtOAc), and *n*-butanol (BuOH) as four organic solvents with increasing polarity. As a result, four main solvent fractions were obtained: *n*-hexane, CH_2_Cl_2_, EtOAc, and BuOH-soluble fractions. Based on the data from LC/MS and thin-layer chromatography (TLC) analysis for the four solvent fractions where major peaks characteristic of fatty acid derivatives were observed in CH_2_Cl_2_-soluble fraction, the CH_2_Cl_2_ fraction was subjected to chemical analysis since the fatty acid derivatives from *O. japonicus* have rarely been investigated in terms of their chemical constituents. The chemical analysis using sequential column chromatography, as well as preparative and semi-preparative HPLC, resulted in the isolation of five fatty acid derivatives (**1**–**5**) (Figure 1).

### 2.2. Structural Elucidation of the Isolated Compounds ***1–5***

Compound **1** was isolated as a colorless oil. The molecular formula was established as C_18_H_30_O_4_ from the molecular ion peak [M + H]^+^ at *m/z* 311.2214 (calcd. for C_18_H_31_O_4_, 311.2222) in the positive-ion mode of the HR-ESIMS. As shown in Table 1, the ^1^H NMR spectrum of **1** showed signals for olefinic protons at *δ*_H_ 7.27 (1H, dd, *J* = 15.5, 11.0 Hz), 6.41 (1H, dd, *J* = 15.0, 11.0 Hz), 6.25 (1H, dd, *J* = 15.0, 6.0 Hz), and 6.20 (1H, d, *J* = 15.5 Hz), an oxygenated methine at *δ*_H_ 4.17 (1H, q, *J* = 6.0 Hz), a terminal methyl group at *δ*_H_ 0.91 (3H, t, *J* = 7.0 Hz), deshielded methylenes at *δ*_H_ 2.62 (2H, t, *J* = 7.5 Hz) and 2.27 (2H, t, *J* = 7.5 Hz), and overlapping signals corresponding to the remaining methylenes from 1.33 to 1.60 ppm. The ^13^C NMR data of **1** (Table 1) obtained by the aid of heteronuclear single quantum coherence (HSQC) spectrum showed four olefinic carbons (*δ*_C_ 147.0, 142.8, 128.9, and 127.3), an oxygenated carbon (*δ*_C_ 71.1), a terminal methyl carbon (*δ*_C_ 13.0), and the remaining carbons attributable to methylenes (*δ*_C_ 22.2 to 39.5). The above NMR data provided sufficient evidence to show that compound **1** is a fatty acid derivative [20].

**Figure 2:**
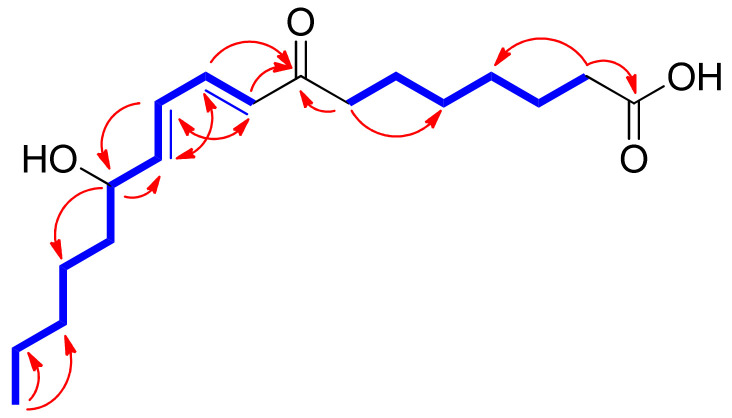
TOCSY (bold lines) and key HMBC (arrows) correlations of **1**.

With the splitting patterns of the olefinic protons, the location of the olefinic groups as well as the hydroxyl group could be estimated as shown in Figure 1, and they were determined to be *trans*-orientated based on the *J* values of 15.0 Hz and 15.5 Hz [20]. This was also confirmed by HMBC correlations of H-9/C-11 (*δ*_C_ 127.3), H-10/C-12 (*δ*_C_ 147.0), H-11/C-9 (*δ*_C_ 128.9) and C-13 (*δ*_C_ 71.1), and H-12/C-10 (*δ*_C_ 142.8), as well as the spin systems observed in the total correlation spectroscopy (TOCSY) spectrum for C-9‒C-10‒C-11‒C-12‒C-13 (Figure 2). The HMBC correlations of H-10/C-8 (*δ*_C_ 202.2), H-9/C-8, and C-7 (*δ*_C_ 39.5) determined a carbonyl group at C-8, and those of H_2_-2/C-1 (*δ*_C_ 177.0) and C-4, H_2_-7/C-5, H_3_-18/C-17, and C-16, along with cross-peaks observed in the TOCSY spectrum for the spin systems of C-2‒C-3‒C-4‒C-5‒C-6‒C-7 and C-13‒C-14‒C-15‒C-16‒C-17‒C-18 determined the remaining gross structure of **1** (Figure 2). The characterized chemical structure of **1** was confirmed by MS/MS analysis, where the MS^2^ of **1** yielded *m/z* 293.2, 157.0, and 101.0 (Figure S7).

**Figure 3:**
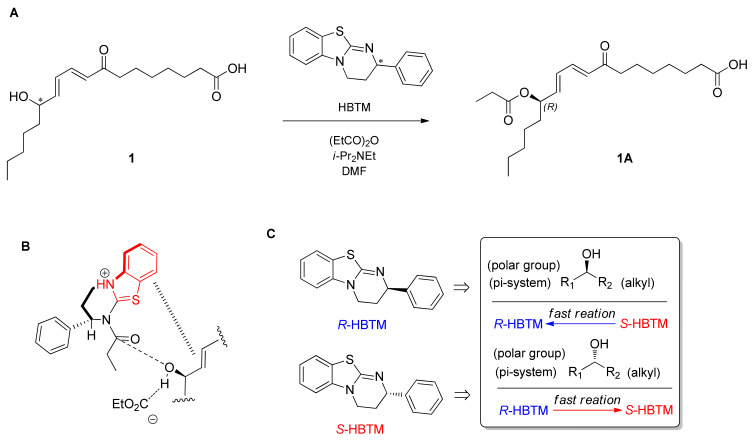
(**A**) CEA reaction for the determination of absolute configuration of compound **1**. (**B**) Proposed favorable transition state of compound **1** in the reaction. (**C**) Mnemonic to predict the configuration of secondary alcohols in CEA reaction. * defines the chiral center.

To assign the absolute configuration of the hydroxyl group at C-13, chemical-derivative method developed by our group, using competing enantioselective acylation (CEA) coupled with LC/MS analysis [21], was utilized. The rates of parallel reactions with the homobenzotetramisole (HBTM) catalysts were compared using LC/MS. Two sets of compound **1** (each 0.2 mg) and *S*- and *R*-HBTM catalysts (each 0.1 mg) were reacted for each parallel acylation reaction. Samples of each reaction were quantitatively analyzed using LC/MS to measure the reaction rate catalyzed by *S*- and *R*-HBTM. The acylated derivative (**1A**, [M + Na]^+^ peak at *m/z* 389), esterified by propionic anhydride in the hydroxyl group at C-13, was expected as a result of the CEA reaction (Figure 3). The anticipated derivatives were detected in samples of both parallel reactions, and the esterification reaction with *S*-HBTM was faster than that with *R*-HBTM (Figure 3; Figure S8), suggesting that compound **1** has a 13*R*-configuration according to the mnemonic to predict the configuration of secondary alcohols in the CEA reaction (Figure 3). Accordingly, the complete structure of compound **1** was determined to be (13*R*)-8-oxo-octadeca-(9*E*,11*E*) dienoic acid and named omphalotol A.

Compound **2** was obtained as a colorless oil. The molecular formula was determined to be C_19_H_34_O_5_ from the molecular ion peak [M − H]^−^ at *m/z* 341.2294 (calcd. for C_19_H_33_O_5_, 341.2328) in the negative-ion mode of HR-ESIMS. The ^1^H NMR spectrum of **2** (Table 1) showed signals of a pair of olefinic protons at *δ*_H_ 6.81 (1H, dd, *J* = 16.0, 5.0 Hz) and 6.38 (1H, dd, *J* = 16.0, 1.5 Hz), two oxygenated methines at *δ*_H_ 4.32 (1H, ddd, *J* = 5.0, 3.5, 1.5 Hz) and 3.79 (1H, m), a methoxyl group at *δ*_H_ 3.67 (3H, s), a terminal methyl group at *δ*_H_ 0.90 (3H, t, *J* = 7.2 Hz), deshielded methylenes at *δ*_H_ 2.57 (2H, t, *J* = 7.4 Hz) and 2.30 (2H, t, *J* = 7.5 Hz), and overlapping signals attributable to the remaining methylenes from 1.31 to 1.62 ppm. The ^13^C NMR data of **2** (Table 1) obtained by the assistance of the HSQC spectrum exhibited two olefinic carbons (*δ*_C_ 142.6, 130.4), two oxygenated carbons (*δ*_C_ 74.2, 74.0), a methoxyl carbon (*δ*_C_ 51.3), a terminal methyl carbon (*δ*_C_ 13.9), and the rest of the carbon signals attributable to methylenes (*δ*_C_ 22.5 to 40.7).

**Figure 4:**
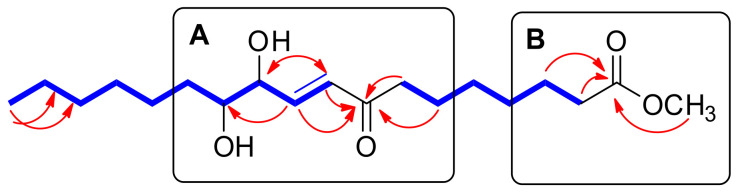
^1^H-^1^H COSY (bold lines) and key HMBC (arrows) correlations of **1**.

The coupling constants of the olefinic protons of H-9 (*J* = 16.0, 1.5 Hz), H-10 (*J* = 16.0, 5.0 Hz), and the oxygenated methine of H-11 (*J* = 5.0, 3.5, 1.5 Hz) supported the *E* configuration of the double bond and the existence of one hydroxyl group next to the olefinic group (Figure 1). The HMBC correlations of H-9/C-11 (*δ*_C_ 74.2), H-9/C-8 (*δ*_C_ 200.5), H-10/C-8, H-10/C-11, H-10/C-12 (*δ*_C_ 74.0), and H-11/C-9 (*δ*_C_ 130.4), as well as spin systems of C-9‒C-10‒C-11‒C-12‒C-13 observed in the ^1^H-^1^H COSY spectrum (Figure 4) further support the above prediction and complete the “A” partial structure of **2**, as shown in Figure 4. The HMBC correlations of 1-OCH_3_/C-1 (*δ*_C_ 174.3), H_2_-2/C-1, and H_2_-3/C-1 determined another “B” partial structure of **2** (Figure 4). The complete planar structure of **2**, including the locations of the olefinic and hydroxyl groups, was determined by MS/MS analysis, where the MS^2^ of **2** yielded *m/z* 241.1 and 171.1 (Figure S15).

**Figure 5:**
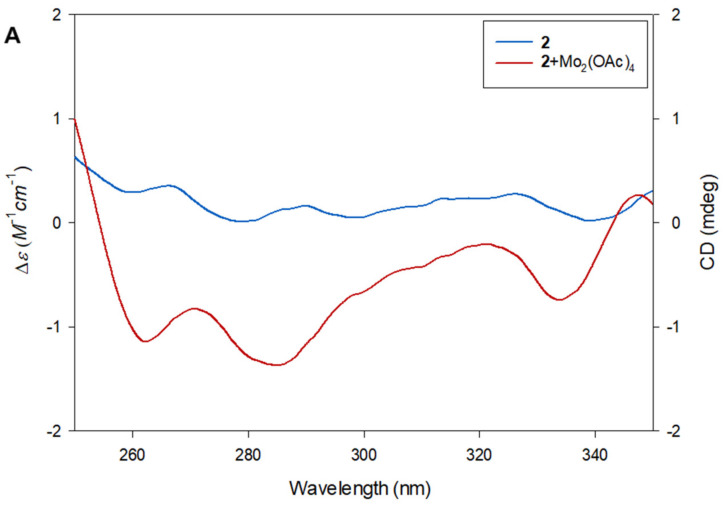

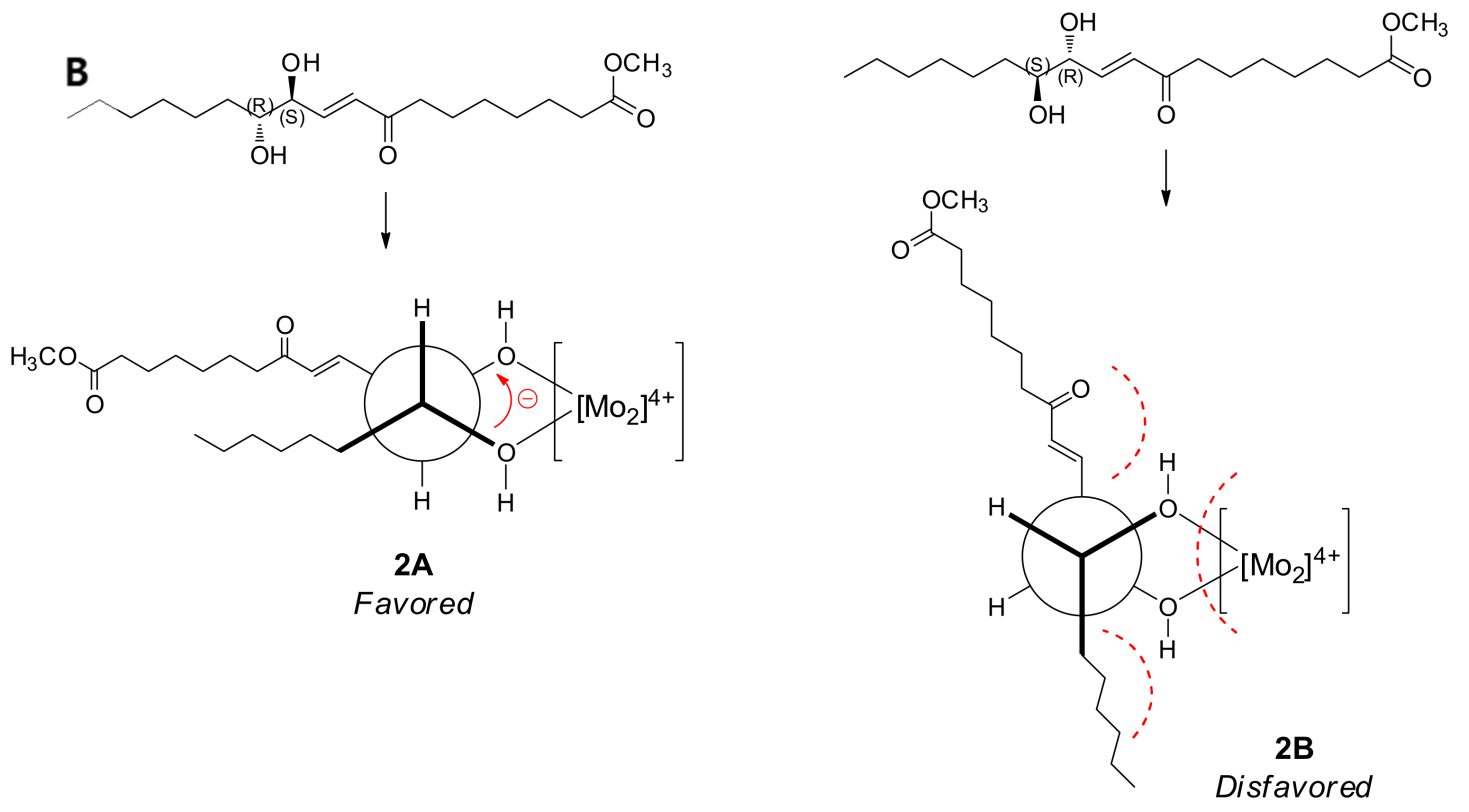
Determination of absolute configurations of C-11 and C-12 in compound **2** according to Snatzke’s method. (**A**) ECD spectrum of **2** and induced ECD spectrum of in situ formed Mo-complex of **2** recorded in DMSO. (**B**) Favored conformations of Mo-complex of **2**.

The absolute configurations of C-11 and C-12 were determined by employing Snatzke’s method [22,23] and the observed *J* value. The small coupling constant (*J* = 3.5 Hz) between H-11 and H-12 indicated the *erythro* configuration of the vicinal diol in **2** [24]. To assign the absolute configuration, a ligand–metal complex was generated by mixing compound **2** and dimolybdenum tetraacetate [Mo_2_(OAc)_4_] as an auxiliary chromophore, for which the induced circular dichroism (ICD) spectrum was recorded [25]. Based on Snatzke’s rule, the Cotton effect at approximately 310 nm reflects the torsional angle of the O‒C‒C‒O moiety of a 1,2-diol derivative in the [Mo_2_(OAc)_4_]-ICD spectrum [22]. As shown in Figure 5, the negative ICD at 310 nm corresponds to a negative torsional angle of the O‒C‒C‒O moiety in the favored conformation (**2A**), which allowed the assignment of the (11*S*,12*R*)-form among the possible *erythro* configurations, (11*S*,12*R*) or (11*R*,12*S*). Compound **2** was characterized as methyl (11*S*,12*R*)-8-oxo-(9*E*)-octadecenoate and named omphalotol B (Figure 1).

The known compounds were identified as (8*E*,10*E*)-7,12-dioxo-8,10-octadecadienoic acid (**3**) [26], pinellic acid (**4**) [27] and methyl (9*S*,12*S*,13*S*)-trihydroxy-10*E*-octadecenoate (**5**) [28], by comparing their NMR spectroscopic and physical data with those previously reported, along with LC/MS analysis.

### 2.3. Antibacterial Activity Evaluation of Isolated Compounds against H. pylori

*H. pylori* is a Gram-negative and microaerophilic bacterium, which causes major public health problems worldwide, affecting approximately 50% of the global population [29]. Eradication of *H. pylori* leads to resolution of both gastritis and gastric ulcers, and even gastric cancer [30]. Although a combination prescription of antibiotics with a proton pump inhibitor is effective, the efficacy has decreased mainly due to the increasing resistance of *H. pylori* strains against antibiotics such as clarithromycin and metronidazole [31,32,33,34]. Therefore, there has been a pressing need to look for new compounds, which can overcome this resistance and provide an effective therapy against *H. pylori* infection. Natural products with less adverse effects can be alternative approaches for the intervention of gastric disorders caused by this bacterium. The isolated compounds **1**–**5** were tested for their antibacterial activity against *H. pylori* strain 51 at the final concentration of 100 μM (Table 2). Among the isolates, compound 1 showed moderate antibacterial activity against *H. pylori* strain 51 with 27.4% inhibition, comparable to that of quercetin as a positive control. Specifically, compound 3 exhibited the most significant antibacterial activity against the strain with 97.5% inhibition (Table 2). Its inhibitory activity, with the minimal inhibitory concentrations (MIC)_50_ and MIC_90_ values of 9 and 20 μM, respectively, was more potent than those of a positive control and metronidazole (MIC_50_ = 17 μM and MIC_90_ = 46 μM). In addition, the minimum bactericidal concentration (MBC) values of compound 3 and metronidazole were 12.5 and 12.5 μM, respectively. The other compounds failed to show anti-*H. pylori* activity. Based on these findings, it is suggested that the α,β-unsaturated carbonyl moiety of compound 3 can play a role in the inhibition of *H. pylori* growth, and the hydroxyl group of compound 2 may decrease the activity. *H. pylori* produces a urease which catalyzes the hydrolysis of urea to produce ammonia for neutralizing the acidic condition of the stomach. It has been known that simple α,β-unsaturated ketones inhibited urease activity by binding to the cysteinyl residue in the active sites of the enzyme [35]. Further study is required to elucidate the exact mechanism of compound 3 to inhibit the growth of *H. pylori*. Specificity to *H. pylori* and toxicity to other cells of this compound are also required in the following study.

## 3. Materials and Methods

### 3.1. General Experimental Procedure

The information on general experimental procedure is provided in Supplementary Materials.

### 3.2. Mushroom Material

Fresh fruiting bodies of *O. japonicus* were collected from Pocheon, Gyeonggi-do, Korea in September 2019. This material was identified by DNA analysis, depending on the modified method [36]. The fungal-specific PCR primers ITS1 and ITS4 were used to amplify the internal transcribed spacer (ITS) region according to a modified method [37]. This sequence homology corresponded to that of *O. japonicus* (syn. *Omphalotus guepiniiformis*)*,* with the highest matching score in the NCBI BLAST network server. A voucher specimen (SKKU-HK-2019-09) of the mushroom was deposited at the herbarium of the School of Pharmacy, Sungkyunkwan University, Korea.

### 3.3. Extraction of O. japonicus and Isolation of Compounds

The dried fruiting bodies of *O. japonicus* (0.6 kg) were extracted with 80% aqueous MeOH three times (each 3 L × 24 h) at room temperature. The resultant extracts were filtered, and the filtrate was evaporated under reduced pressure using a rotary evaporator to obtain a crude MeOH extract (43.6 g). The extract was suspended in distilled water (700 mL) and MeOH (30 mL) and successively solvent-partitioned three times with *n*-hexane, dichloromethane, ethyl acetate, and *n*-butanol, yielding soluble layers of *n*-hexane (6.3 g), CH_2_Cl_2_ (6.7 g), EtOAc (2.4 g), and *n*-butanol (15.6 g). The CH_2_Cl_2_ fraction (6.7 g) was subjected to silica gel column chromatography (CC) (a gradient solvent system; CH_2_Cl_2_/MeOH, from 70:1 to 1:1) to yield five fractions (Fr. C1–C5). Fr. C3 (1.2 g) was subjected to reverse phase (RP) C_18_ CC to yield seven subfractions (Fr. C31–C37). Fr. C34 (91.2 mg) was purified by semi-preparative HPLC (MeOH/H_2_O, 67:33) to give compounds **5** (*t_R_* 19.0 min, 2.2 mg), **1** (*t_R_* 23.5 min, 3.6 mg), **2** (*t_R_* 37.0 min, 1.3 mg), and **3** (*t_R_* = 39.5 min, 0.3 mg). Fr. C4 (1.7 g) was subjected to RP-C_18_ CC, yielding eight subfractions (Fr. C41–C48). Fr. C41 (203.4 mg) was fractionated using preparative HPLC (a gradient solvent system; MeOH/H_2_O, from 50:50 to 100:0), which yielded five subfractions (Fr. C411–C415). Fr. C413 (61.5 mg) was purified using semi-preparative HPLC (MeOH/H_2_O, 54:46) to yield compound **4** (*t_R_* 35.0 min, 1.8 mg).

#### 3.3.1. Omphalotol A (**1**)

Colorless oil; ${\left[\alpha \right]}_{D}^{25}$ −2.7 (*c* 0.07, MeOH); UV (MeOH) *λ*_max_ (log *ε*) 270 (3.4) nm; IR (KBr) *ν*_max_: 3385, 2950, 2831, 1722, 1030 cm^−1^; ^1^H and ^13^C NMR spectroscopic data, see Table 1; (+)-HR-ESIMS *m/z* 311.2214 [M + H]^+^ (calcd. for C_18_H_31_O_4_, 311.2222).

#### 3.3.2. Omphalotol B (**2**)

Colorless oil; ${\left[\alpha \right]}_{D}^{25}$ +30.5 (*c* 0.07, MeOH); UV (MeOH) *λ*_max_ (log *ε*) 215 (2.6) nm; IR (KBr) *ν*_max_: 3371, 2864, 1710, 1018 cm^−1^; ECD (MeOH) λ_max_ (Δε) 245 (+0.3), 268 (+0.3), 292 (+0.1), 328 (+0.3) nm; ^1^H and ^13^C NMR spectroscopic data, see Table 1; (–)-HR-ESIMS *m/z* 341.2294 [M − H]^–^ (calcd. for C_19_H_33_O_5_, 341.2328).

### 3.4. MS/MS Analysis of Compounds ***1*** and ***2***

Stock solutions of compounds **1** and **2** were prepared by dissolving 0.1 mg of sample in 200 μL MeOH. The solution was further diluted with MeOH, filtered through a 0.45-μm hydrophobic PTFE filter, and finally analyzed by LC/MS/MS, Agilent 1290 Infinity II series with 6545 LC/quadrupole time of flight (Q-TOF) mass spectrometer (Agilent Technologies). Analysis was performed by injecting 1 μL of the sample using an Agilent Eclipse Plus C_18_ RRHD (1.8 μm, 2.1 × 50 mm) set at 30 °C. The mobile phase consisting of formic acid in H_2_O (0.1% (*v*/*v*)) (A) and formic acid in acetonitrile (0.1% (*v*/*v*)) (B) was delivered at a flow rate of 0.3 mL/min by applying the following programmed gradient elution: 0–3.0 min, 10% (B); 3.0–10.0 min, 10–100% (B); 10.0–12.0 min, 100% (B); 12.0–15.0 min, 10% (B). The MS system was equipped with an ESI source and operated in both negative and positive ionization modes with a data acquisition range from 100 to 600 *m/z.*

### 3.5. Experimental Procedures to Determine the Absolute Configuration of Compound ***1***

#### 3.5.1. CEA Reaction

Parallel reactions for the CEA reaction were performed as reported by Lee et al. [21], using *S*- and *R*-HBTM. Compound **1** (0.5 mg, 1.61 μmol) was transferred to two transparent, capped 5 mL vials at room temperature, and dimethylformamide (DMF) (90 μL) was added as the organic solvent for the CEA reaction. Both *S*- and *R*-HBTM (10 μL, 0.38 μmol) were added, and *N*,*N*-diisopropylethylamine (1.0 μL, 5.3 μmol) was successively transferred. Propionic anhydride (0.6 μL, 5.3 μmol) was added to start the CEA reaction. After 10 min, 2 μL aliquots from each reaction were acquired for LC/MS analysis and quenched with 98 μL of MeOH to make a total volume of 100 μL.

#### 3.5.2. LC/MS Analysis

An aliquot (5 μL) of the sample (100 μL) acquired from each parallel reaction was directly injected into the LC/MS (Phenomenex Luna C_18_, 4.6 × 100 mm, 3.5 μm, flow rate: 0.3 mL/min; Torrance, CA, USA), and full scans in positive- and negative-ion modes (scan range *m/z* 100−1000) were applied to identify the desired acylated derivative. The mobile phase consisted of 0.1% (*v*/*v*) formic acid in distilled water (A) or acetonitrile (B) with a gradient solvent system as follows: 10−100% B for 10 min, 100% B (isocratic) for 5 min, and then 10% B (isocratic) for 5 min for the post-run washing procedure of the column. The reaction rate catalyzed by both *S*- and *R*-HBTM was determined by measuring the peak areas of the acylated derivatives.

### 3.6. Absolute Configuration of the 1,2-diol Functionalities in Compound ***2***

According to a published procedure [22,23], **2** (0.5 mg) and Mo_2_(OAc)_4_ (0.75 mg) were mixed in 1.0 mL of dry DMSO with a ligand-to-metal molar ratio of approximately 1.0:1.2, and the solution was directly subjected to ECD measurements. The first circular dichroism (CD) spectrum was recorded immediately after mixing, and its time evolution was monitored until it was stationary (approximately 30 min after mixing). The inherent CD was subtracted. The observed signs of the diagnostic band at approximately 310 nm in the induced CD spectra were correlated with the absolute configuration of the 1,2-diol moiety.

### 3.7. Anti-Helicobacter pylori Activity

A clinical strain of *H. pylori* 51 isolated from a Korean patient with a duodenal ulcer (HPKTCC B0006) was provided by the *H. pylori* Korean Type Culture Collection, School of Medicine, Gyeongsang National University, Korea. The strain was grown and maintained on Brucella agar medium (BD Co., Sparks, MD, USA) supplemented with 10% horse serum (Gibco, New York, NY, USA). The culture conditions were 37 °C, 100% humidity, and 10% CO_2_ for 2–3 days.

MICs were determined by the broth dilution method previously reported [38,39]. Twenty microliters of bacterial colony suspension equivalent to 2–3 × 10^8^ cfu/mL and twenty microliters of two-fold diluted samples and controls were added to each well of a 6-well plate containing Brucella broth medium supplemented with 10% horse serum. The final volume was brought to 2 mL. After 24 h of incubation, bacterial growth was evaluated by measuring the optical density at 600 nm on a spectrophotometer (Optizen POP, Mecasys, Daejeon, Korea). MIC_50_ and MIC_90_ values were defined as the lowest concentrations of samples at which bacterial growth was inhibited by 50% and 90%, respectively, and were calculated using GraphPad Version 5.01 (GraphPad Software, Inc., San Diego, CA, USA).

MBC was determined by re-culturing broth dilution that inhibits the growth of *H. pylori* on the agar plate. Twenty microliter of broth dilution was streaked onto Brucella agar plate and incubated for 48 h. The MBC value was defined as the lowest concentration that showed no colonies of bacteria on agar plates.

### 3.8. Statistical Analysis

One-way analysis of variance was performed using Excel 2019 (Microsoft, Redmond, WA, USA). Values with *p* < 0.05 were considered statistically significant.

## 4. Conclusions

In this study, we isolated and identified five fatty acid derivatives (**1**–**5**), including two new fatty acid derivatives, omphalotols A and B (**1** and **2**), from the methanolic extracts of *O. japonicus* fruiting bodies. The structures of the new compounds were established using NMR spectroscopy and LC–MS analysis, as well as fragmentation patterns in MS/MS data and chemical reactions followed by the application of Snatzke’s method and competing enantioselective acylation (CEA). In the anti-*H. pylori* activity test, we demonstrated that compound **1** showed moderate antibacterial activity against *H. pylori* strain 51 comparable to that of quercetin, a positive control. Specifically, compound **3** displayed the most significant anti-*H. pylori* activity with 97.5% inhibition, and its inhibitory activity with MIC_50_ and MIC_90_ values of 9 and 20 μM, respectively, was more potent than those of metronidazole (MIC_50_ = 17 μM and MIC_90_ = 46 μM). Based on these findings, we conclude that compound **3**, an α,β-unsaturated ketone derivative, could be used as a moiety in the development of novel antibiotics against *H. pylori*; however, further studies on its mechanism, antibacterial activity against another species, and toxicity to normal and cancerous cell lines are needed.

## Figures and Tables

**Table 1:** ^1^H and ^13^C NMR data for compounds **1** and **2** (*δ* ppm) ^a^.

Position	1		2	
	*δ*_H_ (*J* in Hz)	δ_C_	*δ*_H_ (*J* in Hz)	*δ*_C_
1		177.0		174.3
2	2.27 t (7.5)	33.9	2.30 t (7.5)	34.0
3	1.60 m ^b^	24.3	1.62 m ^b^	24.4
4	1.35 m ^b^	28.5	1.31 m ^b^	28.8
5	1.35 m ^b^	28.5	1.31 m ^b^	31.6
6	1.60 m ^b^	24.3	1.62 m ^b^	24.4
7	2.62 t (7.5)	39.5	2.57 t (7.5)	40.7
8		202.2		200.5
9	6.20 d (15.5)	128.9	6.38 dd (16.0, 1.5)	130.4
10	7.27 dd (15.5, 11.0)	142.8	6.81 dd (16.0, 5.0)	142.6
11	6.41 dd (15.0, 11.0)	127.3	4.32 ddd (5.0, 3.5, 1.5)	74.2
12	6.25 dd (15.0, 6.0)	147.0	3.79 m	74.0
13	4.17 q (6.0)	71.1	1.43 m	32.1
14	1.54 m	36.5	1.31 m ^b^	28.8
15	1.35 m ^b^	24.8	1.31 m ^b^	25.2
16	1.33 m ^b^	22.2	1.31 m ^b^	22.5
17	1.33 m ^b^	31.7	1.31 m ^b^	31.6
18	0.91 t (7.0)	13.0	0.90 t (7.0)	13.9
1-OCH_3_			3.67 s	51.3

^a^ 700 MHz in CD_3_OD for **1** and 850 MHz in CDCl_3_ for **2**; coupling constants (in Hz) are in parentheses. Assignments were based on the HSQC, HMBC, and TOCSY/^1^H-^1^H COSY spectra. ^b^ Overlapped.

**Table 2:** Anti-*H. pylori* activity of compounds **1**–**5**.

Compound	Concentration (μM)	Inhibition (%)	MIC (μM)	MIC_50_ (μM)	MIC_90_ (μM)
**1**	100	27.4 ± 4.5 ^b^			
**2**		11.1 ± 0.3 ^c^			
**3**		97.5 ± 0.8 ^a^	3.1	9	20
**4**		2.5 ± 0.8 ^d^			
**5**		0.2 ± 0.1 ^d^			
Quercetin *	100	34.4 ± 0.6 ^b^	50		
Metronidazole *		97.0 ± 0.1 ^a^	6.3	17	46

* Positive controls. Data are presented as mean ± SD of experiments in duplicates. Different upper letters in the same column indicate a significant difference *(p* < 0.05) among the samples.

## Data Availability

Not applicable.

## Supplementary Materials

The following are available online at https://www.mdpi.com/article/10.3390/ph15020139/s1; Figure S1: HR-ESIMS data of **1**; Figure S2: UV spectrum of **1**; Figure S3: ^1^H NMR spectrum of **1** (CD_3_OD, 700 MHz); Figure S4: HSQC spectrum of **1**; Figure S5: HMBC spectrum of **1**; Figure S6: TOCSY spectrum of **1**; Figure S7: MS/MS analysis of **1** (MS^2^ 333 [M + Na]^+^ → full-scan); Figure S8: LC/MS data of acylated derivative from CEA reaction of **1**: (A) An acylated derivative of compound **1** in *R*-HBTM catalyzed acylation reaction; (B) An acylated derivative of compound **1** in *S*-HBTM catalyzed acylation reaction; Figure S9: HR-ESIMS data of **2**; Figure S10: UV spectrum of **2**; Figure S11: ^1^H NMR spectrum of **2** (CDCl_3_, 850 MHz); Figure S12: HSQC spectrum of **2**; Figure S13: HMBC spectrum of **2**; Figure S14: ^1^H-^1^H COSY spectrum of **2**; Figure S15: MS/MS analysis of **2** (MS^2^ 341 [M − H]^−^ → full-scan). General experimental procedure.
