# Supplementary material for: Two New Fatty Acid Derivatives, Omphalotols A and B and Anti-Helicobacter pylori Fatty Acid Derivatives from Poisonous Mushroom Omphalotus japonicus
Source: Pharmaceuticals (Basel). 2022 Jan 25;15(2):139. doi: 10.3390/ph15020139 (PMC8874359; doi:10.3390/ph15020139)
Supplement: Supplementary file 1 [file pharmaceuticals-15-00139-s001.zip › pharmaceuticals-1530678-supplementary.pdf]

Supplementary Materials

Two New Fatty Acid Derivatives, Omphalotols A and B and Anti-*Helicobacter pylori* Fatty Acid Derivatives from Poisonous Mushroom *Omphalotus japonicus*

Seulah Lee [1,2,†], Tae Wan Kim [1,†], Yong Hoon Lee [1], Dong-Min Kang [3], Rhim Ryoo [4], Yoon-Joo Ko [5], Mi-Jeong Ahn [3], and Ki Hyun Kim[1,*]

[1] School of Pharmacy, Sungkyunkwan University, Suwon 16419, Korea; seulah@kopri.re.kr (S.L.); asde8282@naver.com (T.W.K.)

[2] Division of Life Sciences, Korea Polar Research Institute, KIOST, Incheon, 21990, Korea

[3] College of Pharmacy and Research Institute of Pharmaceutical Sciences, Gyeongsang National University, Jinju 52828, Korea; kdm7105@gnu.ac.kr (D.M.K.); amj5812@gnu.ac.kr (M.J.A.)

[4] Special Forest Products Division, Forest Bioresources Department, National Institute of Forest Science, Suwon 16631, Korea; rryoo@korea.kr (R.R.)

[5] Laboratory of Nuclear Magnetic Resonance, National Center for Inter-University Research Facilities (NCIRF), Seoul National University, Gwanak-gu, Seoul 08826, Korea; yjko@snu.ac.kr (Y.J.K.)

[*] Correspondence: khkim83@skku.edu (K.H.K.); Tel.: +82-31-290-7700 (K.H.K.)

[†] These authors contributed equally to this study

List of Contents

Figure S1. HR-ESIMS data of **1**……3
Figure S2. UV spectrum of **1**……4
Figure S3. ^{1}H NMR spectrum of **1** (CD_3OD, 700 MHz)……5
Figure S4. HSQC spectrum of **1**……6
Figure S5. HMBC spectrum of **1**……7
Figure S6. TOCSY spectrum of **1**……8
Figure S7. MS/MS analysis of **1** (MS^2 333 $[M + Na]^+$ → full-scan)……9
Figure S8. LC/MS data of acylated derivative from CEA reaction of **1**……10
Figure S9. HR-ESIMS data of **2**……11
Figure S10. UV spectrum of **2**……12
Figure S11. ^{1}H NMR spectrum of **2** ($CDCl_3$, 850 MHz)……13
Figure S12. HSQC spectrum of **2**……14
Figure S13. HMBC spectrum of **2**……15
Figure S14. ^{1}H-^{1}H COSY spectrum of **2**……16
Figure S15. MS/MS analysis of **1** (MS^2 341 $[M – H]^-$ → full-scan)……17
General experimental procedure ……18

Figure S1. HR-ESIMS data of **1**

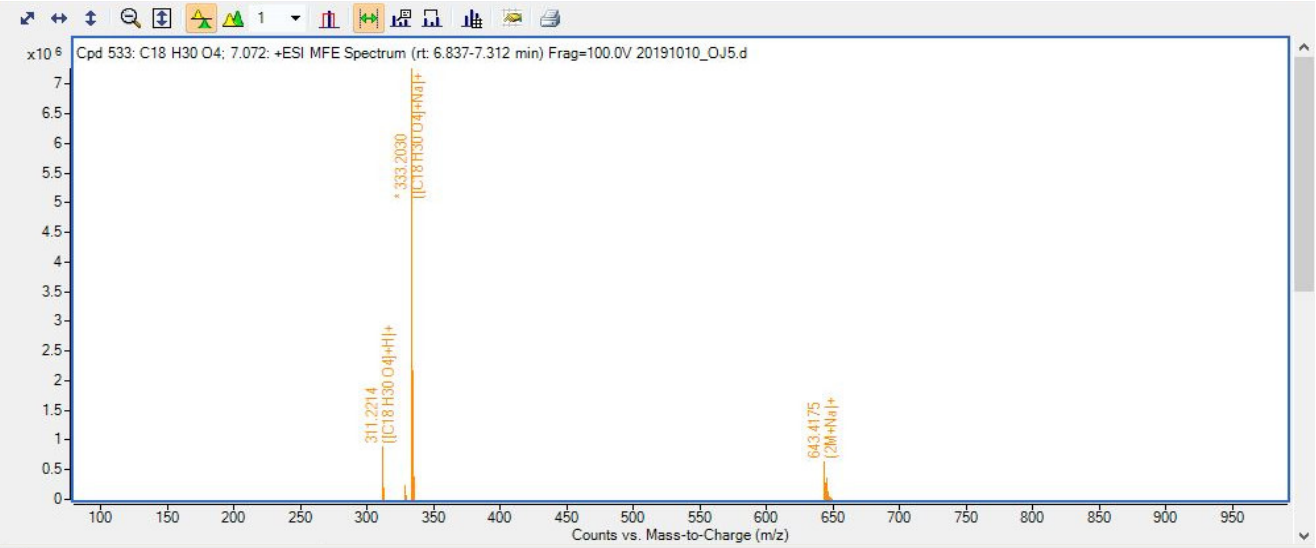

Compound Identification Results: Cpd 10: C18 H30 O4; 7.072 - 20191010_OJ5.d

ID Techniques Applied: MFG

Best	Formula	m/z	Mass	Diff (ppm)	RT	Species	Score
○	C13 H30 N2 O6	333.2030	310.2144	12.89	7.072	(M+Na)+	57.21
⦿	C18 H30 O4	311.2214 333.20...	310.2143	-0.29	7.072	(M+H)+ (M+Na)+	91.56
○	C16 H28 N3 O3	311.2214 333.20...	310.2144	4.26	7.072	(M+H)+ (M+Na)+	85.37
○	C19 H26 N4	311.2214 333.20...	310.2144	-4.38	7.072	(M+H)+ (M+Na)+	81.84
○	C14 H26 N6 O2	311.2214 333.20...	310.2145	8.85	7.072	(M+H)+ (M+Na)+	70.18
○	C21 H28 N O	311.2214 333.20...	310.2143	-8.9	7.072	(M+H)+ (M+Na)+	67.91
○	C12 H24 N9 O	311.2214 333.20...	310.2146	13.47	7.072	(M+H)+ (M+Na)+	53.94

Figure S2. UV spectrum of **1**

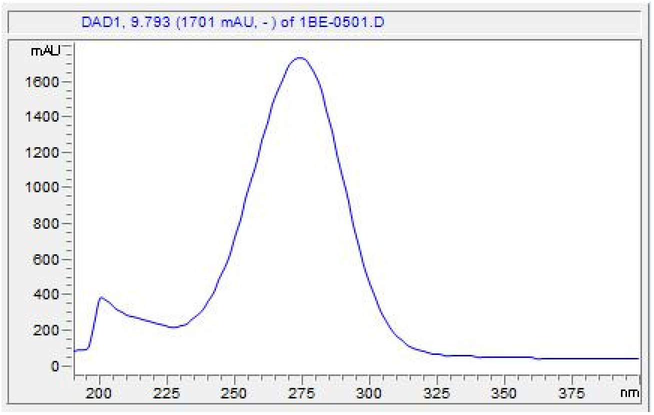

Figure S3. ^{1}H NMR spectrum of **1** (CD_3OD, 700 MHz)

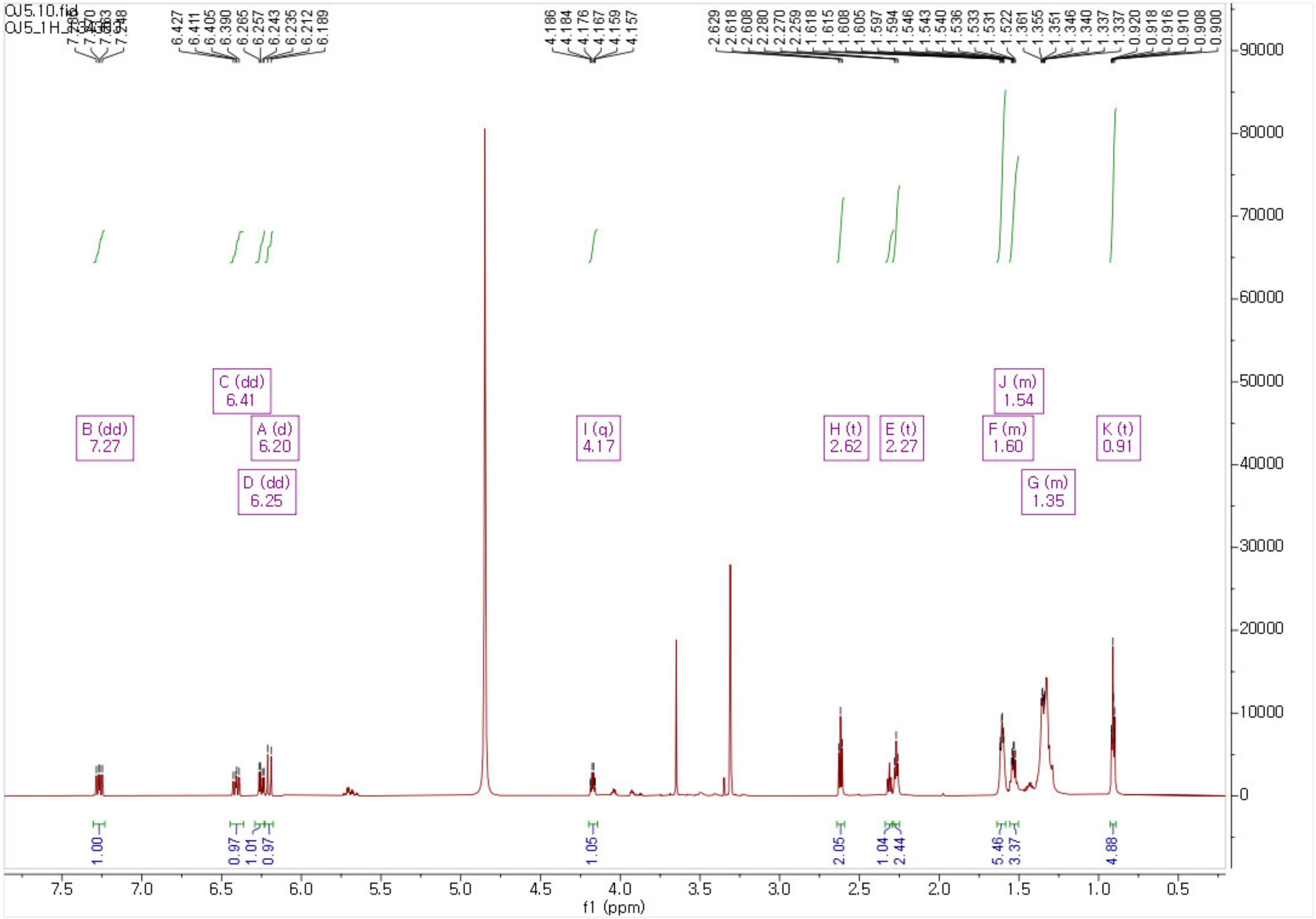

Figure S4. HSQC spectrum of **1**

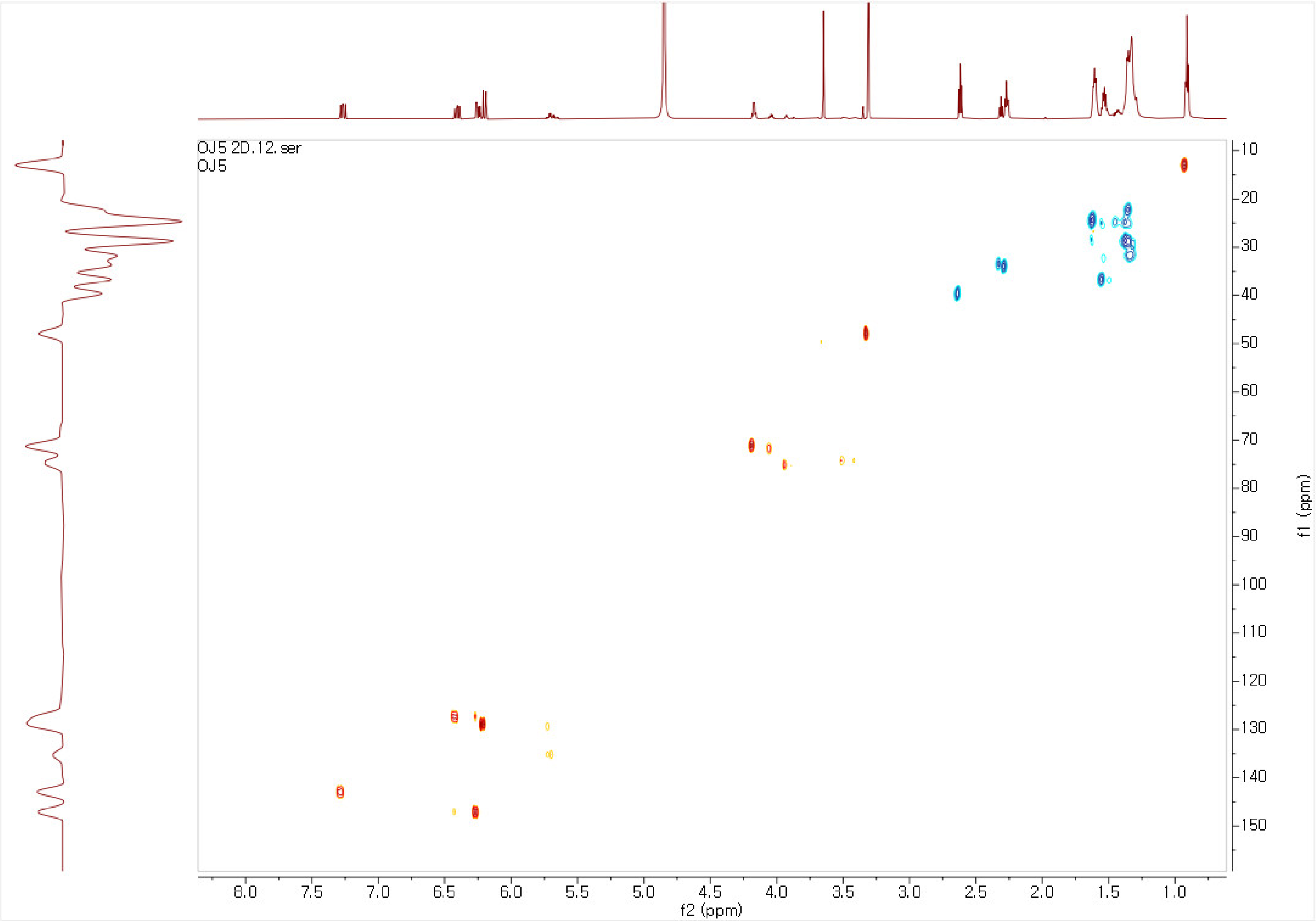

Figure S5. HMBC spectrum of **1**

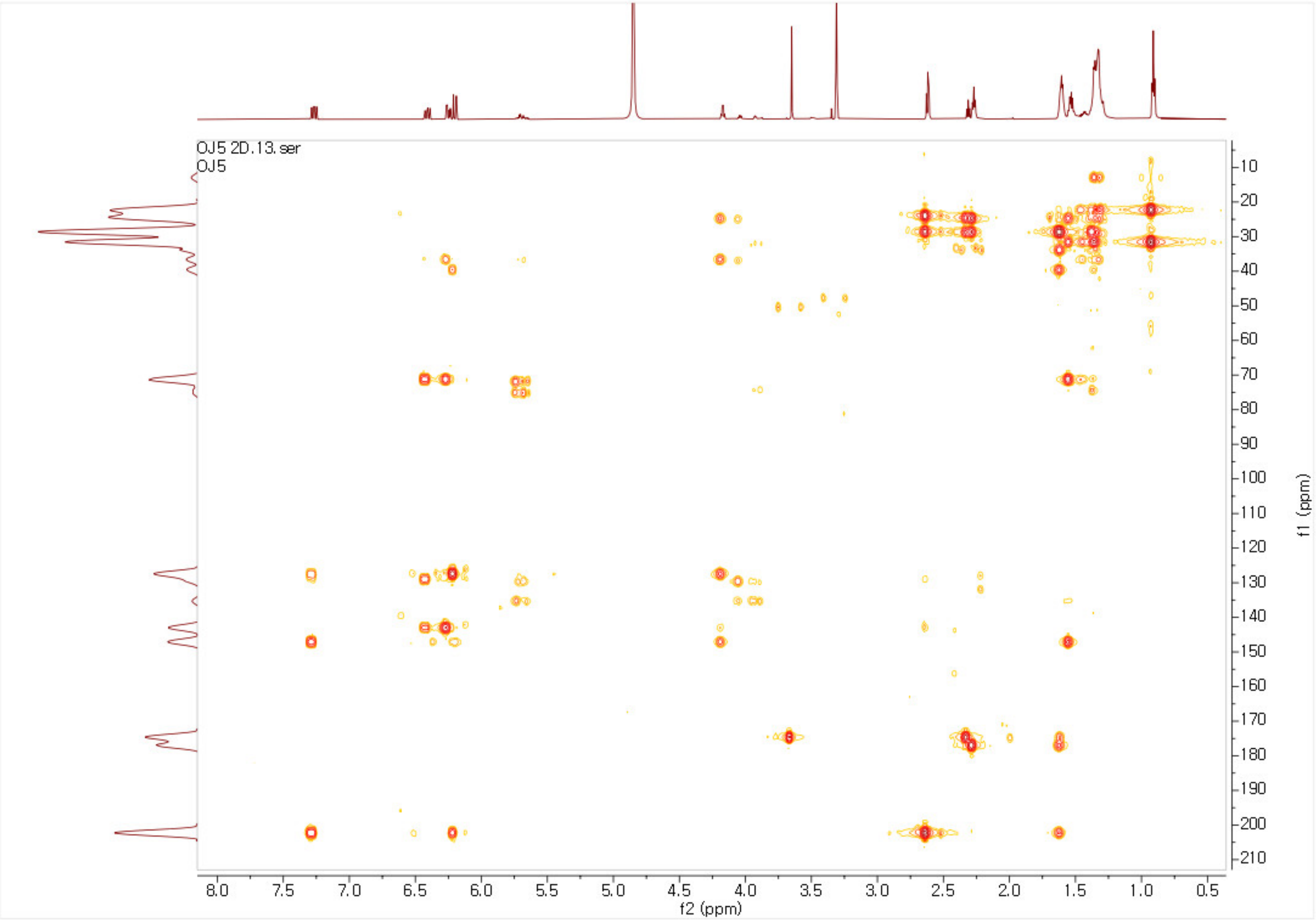

Figure S6. TOCSY spectrum of **1**

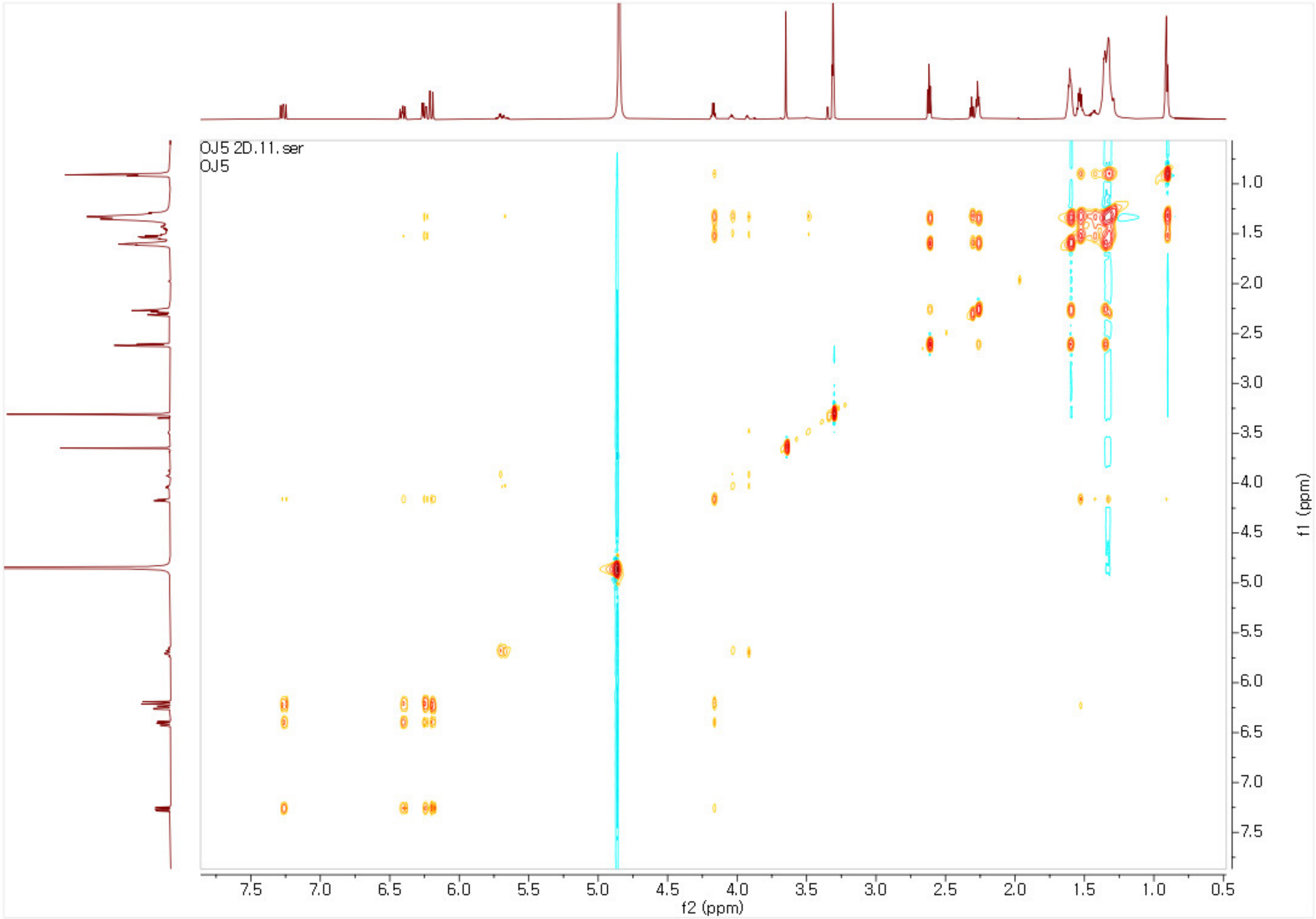

Figure S7. MS/MS analysis of **1** (MS^2 333 $[M + Na]^+$ → full-scan)

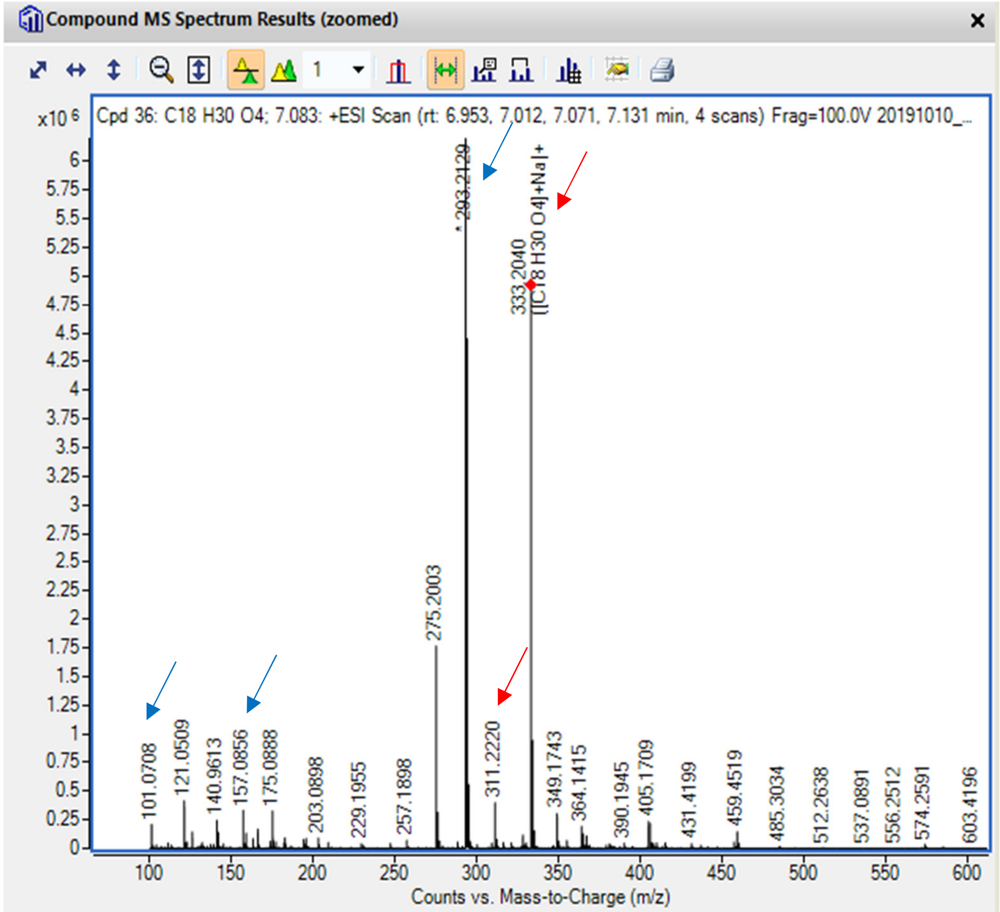

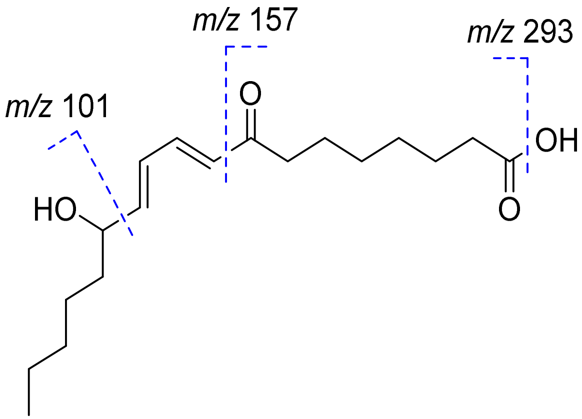

Figure S8. LC/MS data of acylated derivative from CEA reaction of **1**: (A) An acylated derivative of compound **1** in *R*-HBTM catalyzed acylation reaction; (B) An acylated derivative of compound **1** in *S*-HBTM catalyzed acylation reaction

(A)

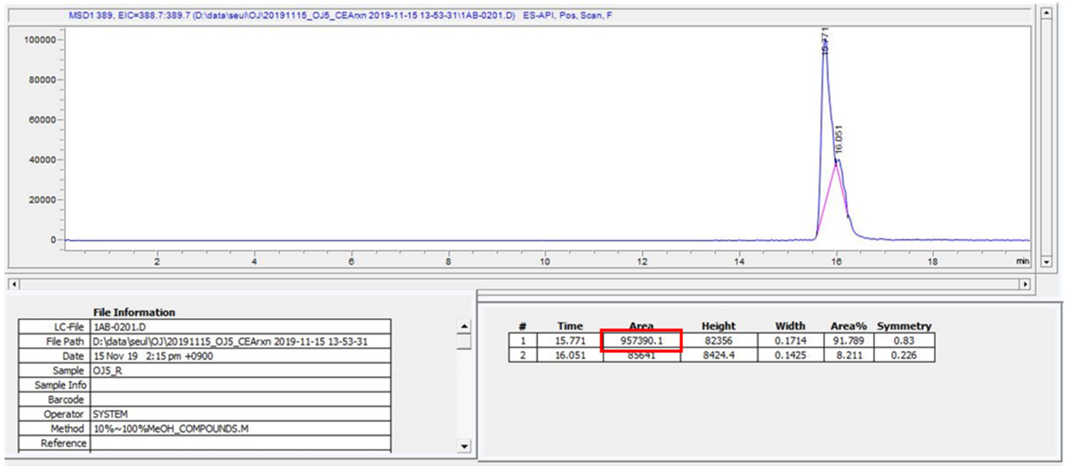

(B)

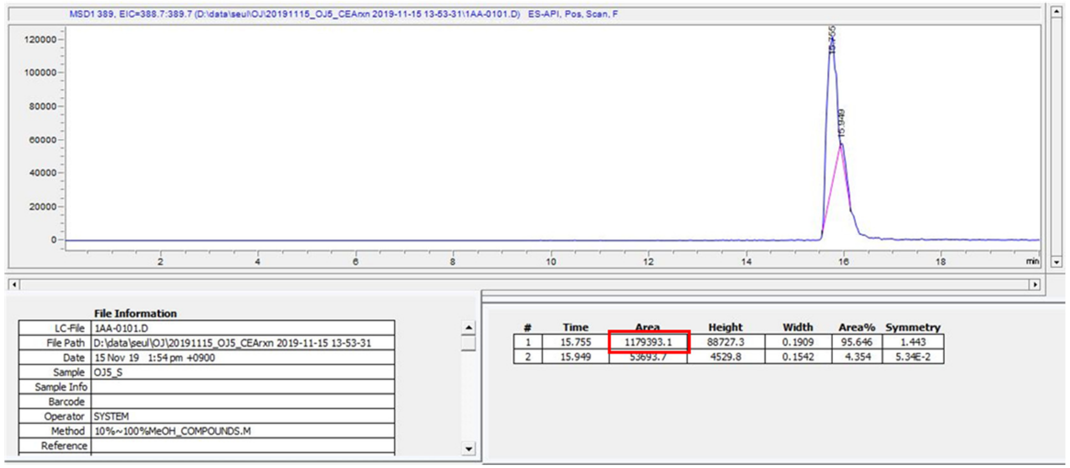

Figure S9. HR-ESIMS data of **2**

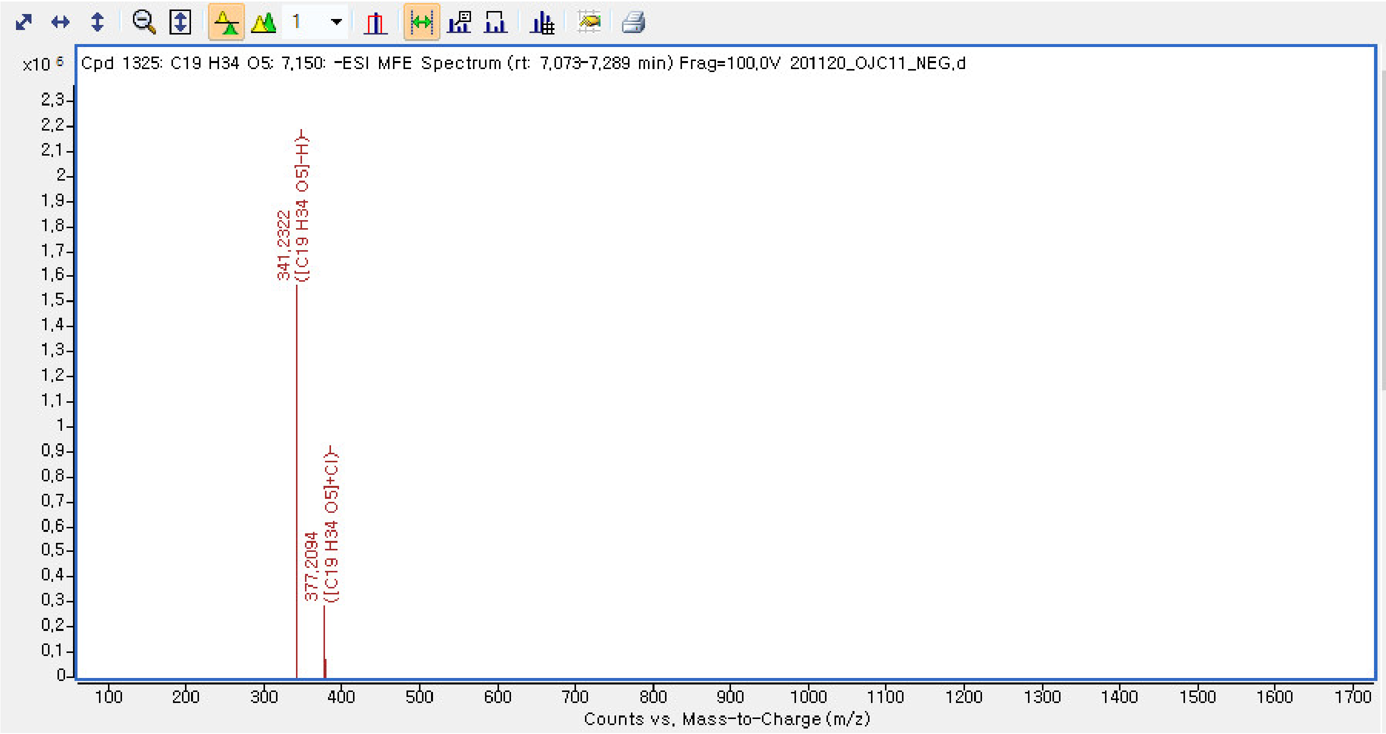

ID Techniques Applied
MFG

Best	Name	Formula	m/z	Mass	Mass (Tgt)	Diff (ppm)	Score (Tgt)	RT	RT (Tgt)	RT Diff	Score (RT)	Species	Score (DB)	Score (Lib)
⊙		C19 H34 O5	341.2322 377.2094	342.2397		-2.74		7.15				(M-H)- (M+Cl)-		

Figure S10. UV spectrum of **2**

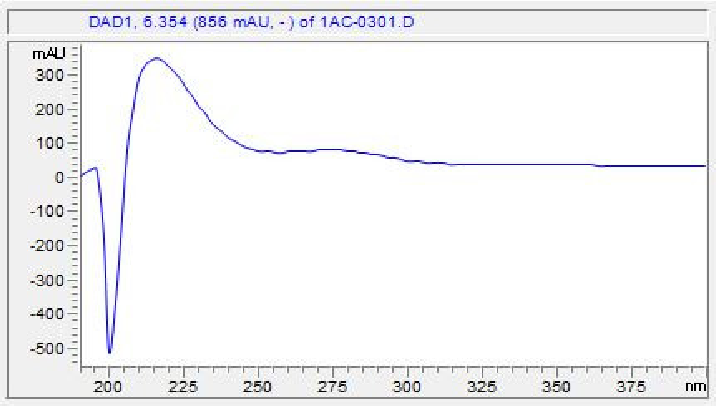

Figure S11. ^{1}H NMR spectrum of **2** ($CDCl_3$, 850 MHz)

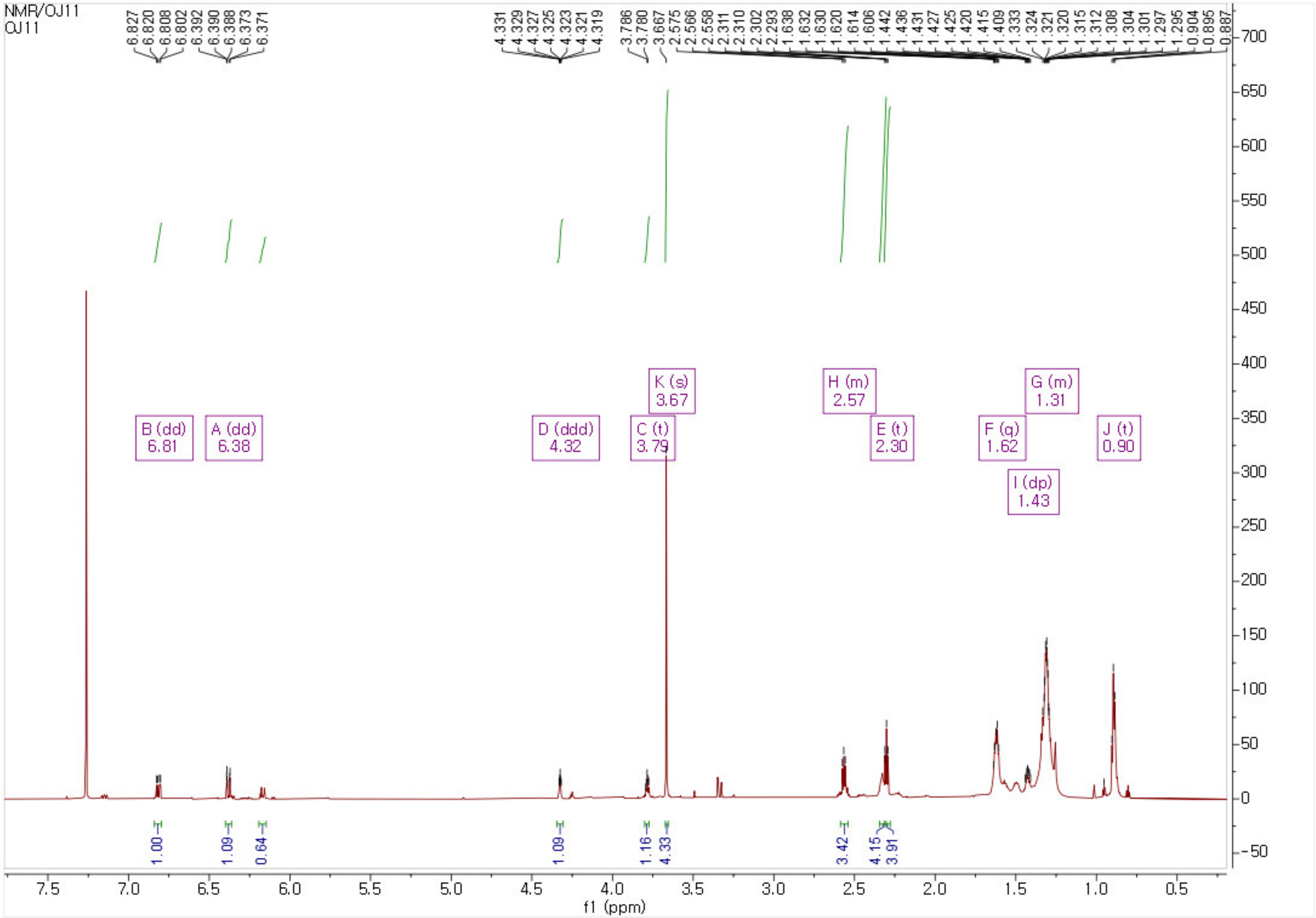

Figure S12. HSQC spectrum of **2**

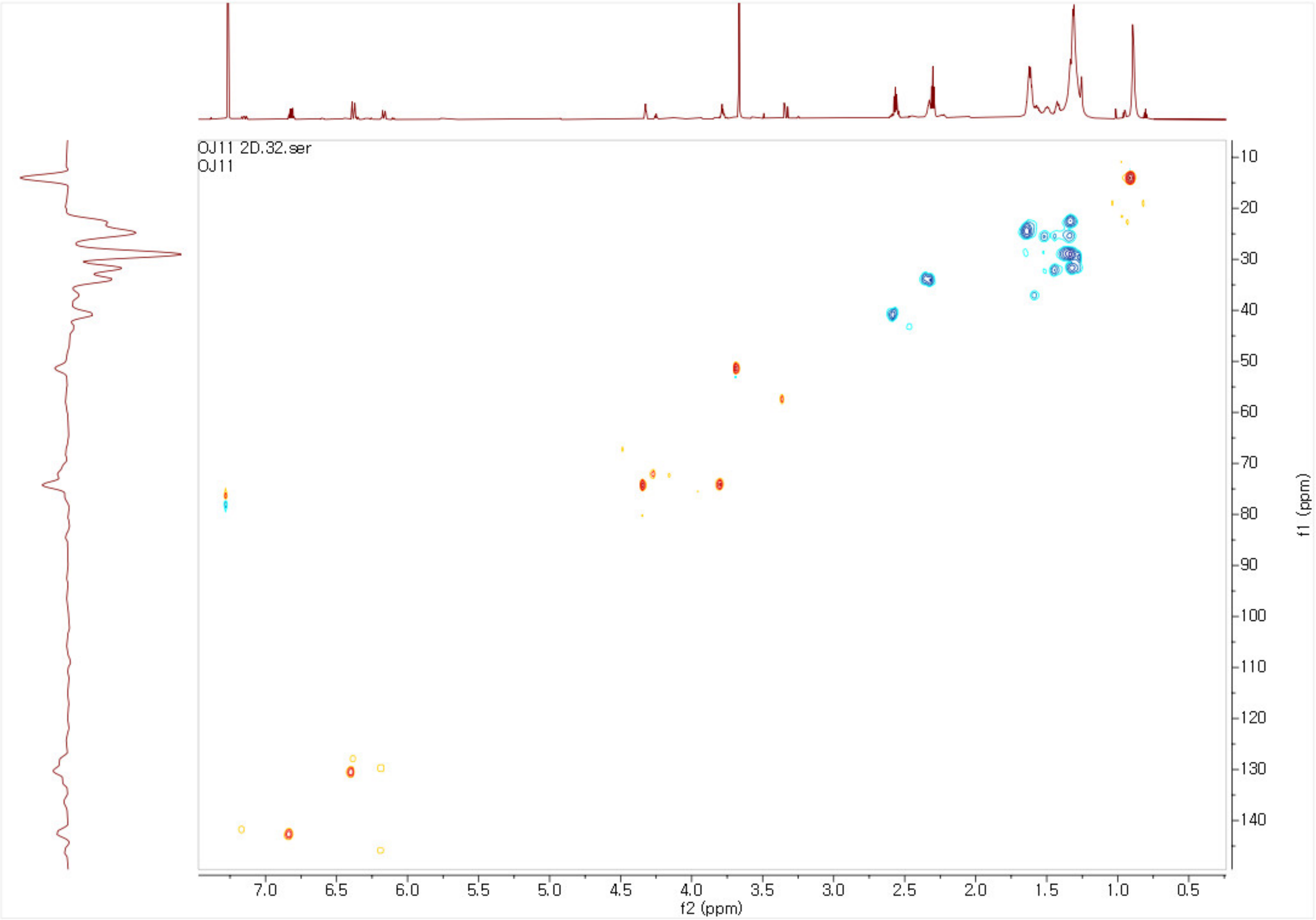

Figure S13. HMBC spectrum of **2**

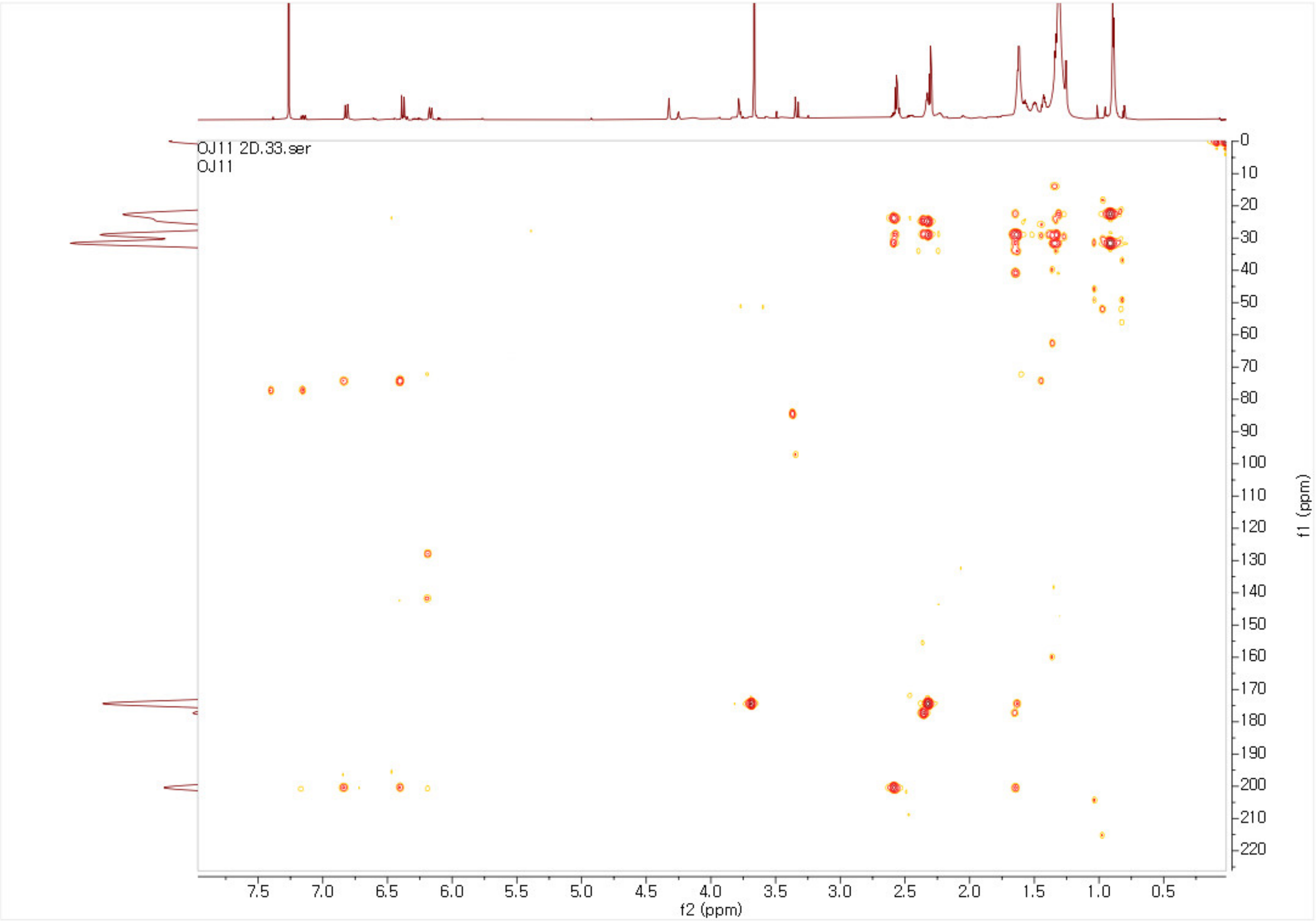

Figure S14. ^{1}H-^{1}H COSY spectrum of **2**

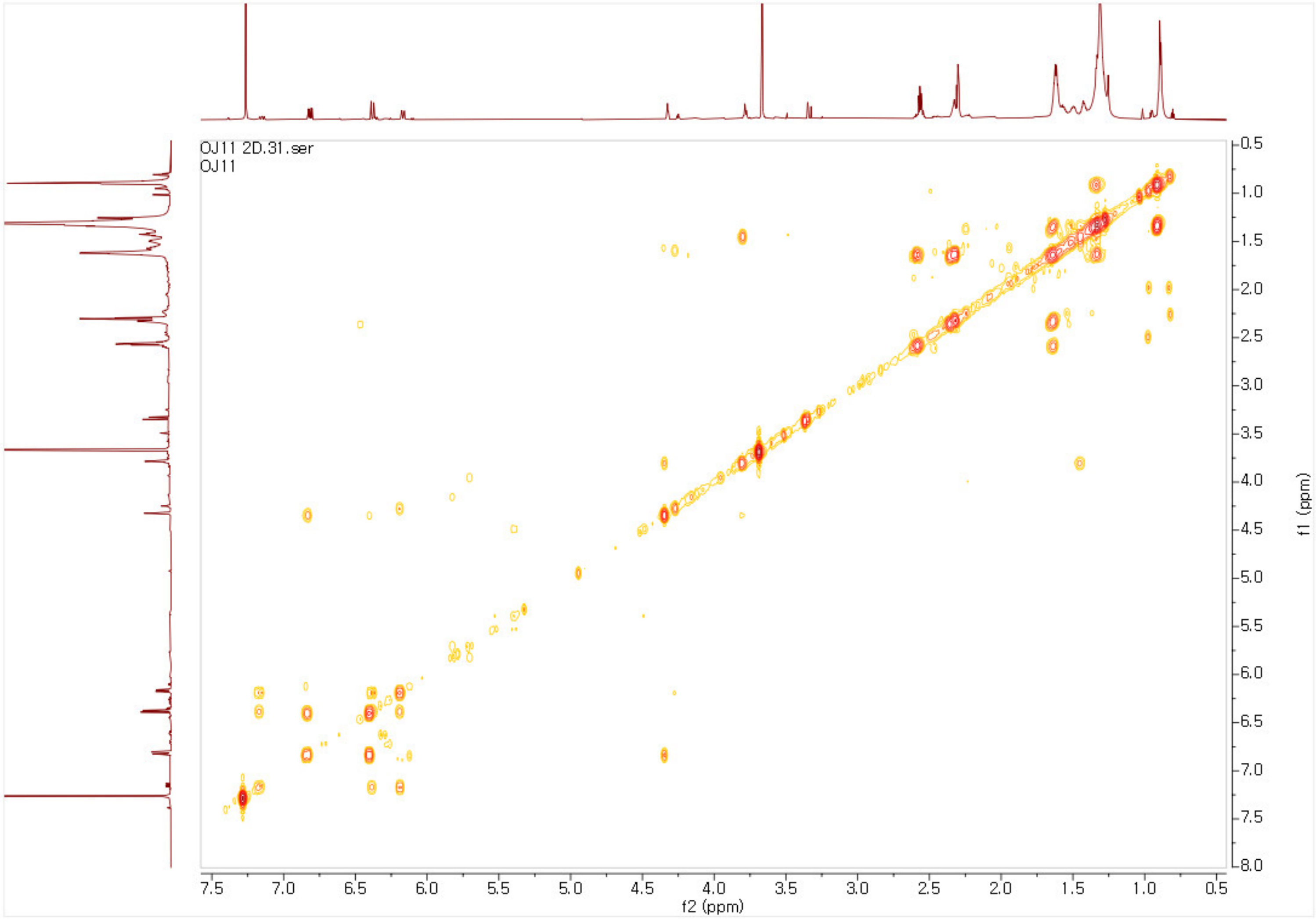

Figure S15. MS/MS analysis of **2** (MS^2 341 $[M - H]^-$ → full-scan)

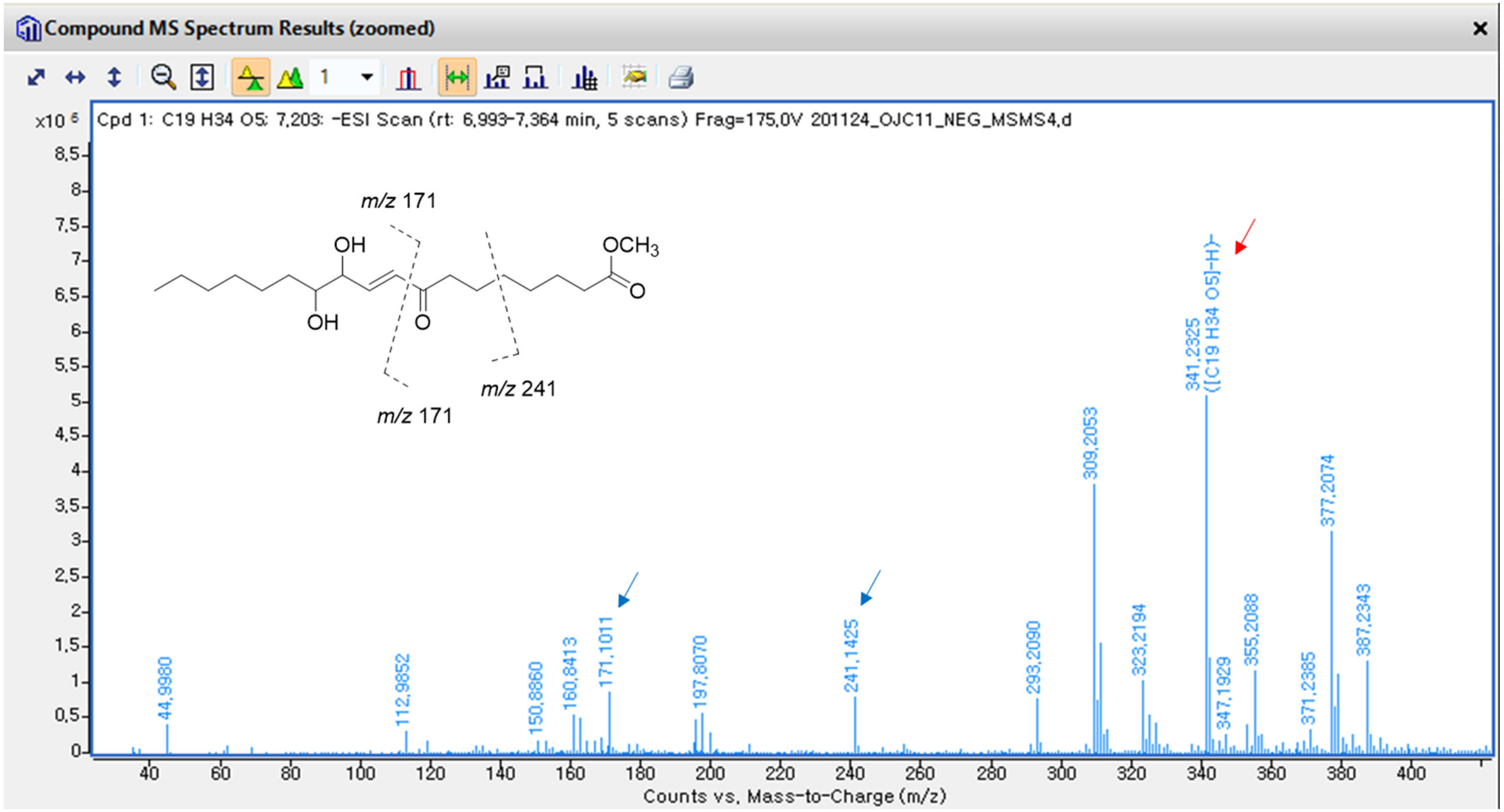

General experimental procedure

Optical rotations were measured using a Jasco P-1020 polarimeter (Jasco, Easton, MD, USA). Infrared (IR) spectra were recorded on a Bruker IFS-66/S FT-IR spectrometer (Bruker, Karlsruhe, Germany). Ultraviolet (UV) spectra were acquired on an Agilent 8453 UV-visible spectrophotometer (Agilent Technologies, Santa Clara, CA, USA). High-resolution (HR)-electrospray ionization (ESI) mass and tandem mass (MS/MS) spectra were recorded on an Agilent 1290 Infinity II series with a 6545 LC/Q-TOF mass spectrometer (Agilent Technologies). NMR spectra were recorded on a Bruker AVANCE III spectrometer (Bruker). Preparative high-performance liquid chromatography (HPLC) was conducted using a Waters 1525 binary HPLC pump with a Waters 996 photodiode array detector (Waters) and an Agilent Eclipse C_{18} column (250 × 21.2 mm, 5 μm; flow rate: 5 mL/min) (Agilent Technologies), and semi-preparative HPLC was performed using a Shimadzu Prominence HPLC System with SPD-20A/20AV Series Prominence HPLC UV-Vis Detectors (Shimadzu, Tokyo, Japan). LC/MS analysis was performed on an Agilent 1200 series HPLC system with a diode array detector and 6130 Series ESI mass spectrometer using an analytical Kinetex C18 100 Å column (100 mm × 2.1 mm i.d., 5 μm) (Phenomenex, Torrance, CA). Column chromatography was performed using silica gel 60 (Merck, Darmstadt, Germany, 230-400 mesh) and RP-C_{18} silica gel (Merck, 230-400 mesh). Thin-layer chromatography (TLC) was conducted using precoated silica gel F_{254} plates and reverse-phase (RP)-18 F_{254s} plates (Merck). Spots on TLC were detected using UV light and heating after dipping in anisaldehyde-sulfuric acid.
